# Effects of esculentoside A on turnour necrosis factor production by mice peritoneal macrophages

**DOI:** 10.1155/S0962935192000565

**Published:** 1992

**Authors:** Fang Jun, Zheng Qin Yue, Wang Hong Bin, Ju Dian Wen, Yi Yang Hua

**Affiliations:** Department of Pharmacology School of Pharmacy Second Military Medical University Shanghai 200433 China

## Abstract

Esculentoside A (EsA) is a saponin isolated from the roots of Phytolacca esculenta. Previous experiments showed that it had strong anti-inflammatory effects. Tumour necrosis factor (TNF) is an important inflammatory mediator. In order to study the mechanism of the anti-inflammatory effect of EsA, it was determined whether TNF production from macrophages was altered by EsA under lipopolysaccharide (LPS) stimulated conditions. EsA was found to decrease both extracellular and cell associated TNF production in a dose dependent manner at concentrations higher than 1 μmol/l EsA. Previous studies have showed that EsA reduced the releasing of platelet activating factor (PAF) from rat macrophages. The reducing effects of EsA on the release of TNF and PAF may explain its anti-inflammatory effect.


Mediators of Inflammation 1, 375–377 (1992)

### Effects of esculentoside A on tumour necrosis factor production by mice peritoneal macrophages

**Fang Jun,[CA] Zheng Qin Yue, Wang Hong Bin, Ju Dian Wen and Yi Yang Hua**

Department of Pharmacology, School of Pharmacy, Second Military Medical University, Shanghai 200433, China

[CA] Corresponding Author

**ESCULENTOSIDE A (EsA) is a saponin isolated from the roots of *Phytolacca esculenta*. Previous experiments showed that it had strong anti-inflammatory effects. Tumour necrosis factor (TNF) is an important inflammatory mediator. In order to study the mechanism of the anti-inflammatory effect of EsA, it was determined whether TNF production from macrophages was altered by EsA under lipopolysaccharide (LPS) stimulated conditions. EsA was found to decrease both extracellular and cell associated TNF production in a dose dependent manner at concentrations higher than 1 μmol/l EsA. Previous studies have showed that EsA reduced the releasing of platelet activating factor (PAF) from rat macrophages. The reducing effects of EsA on the release of TNF and PAF may explain its anti-inflammatory effect.**

**Key words:** Esculentoside A, macrophage, tumour necrosis factor

### Introduction

Esculentoside A (EsA) is a saponin isolated from the root of *Phytolacca esculenta*, and is identified as: 3-O-[β-D-glucopyranosyl-(1-4)-β-D-xylopyranosyl] phytolaccagenin. The structure of this compound is shown in Fig. 1. Experiments showed that it has strong anti-inflammatory effects.[1] It is now known that tumour necrosis factor (TNF) possesses a number of properties of inflammatory response.[2] Platelet activating factor (PAF) is also an inflammatory mediator.[3] It has been shown that EsA inhibited the production of PAF by A23187 stimulated rat macrophages.[4] The aim of this work was to evaluate the effects of EsA on the production of TNF by LPS stimulated mice peritoneal macrophages.

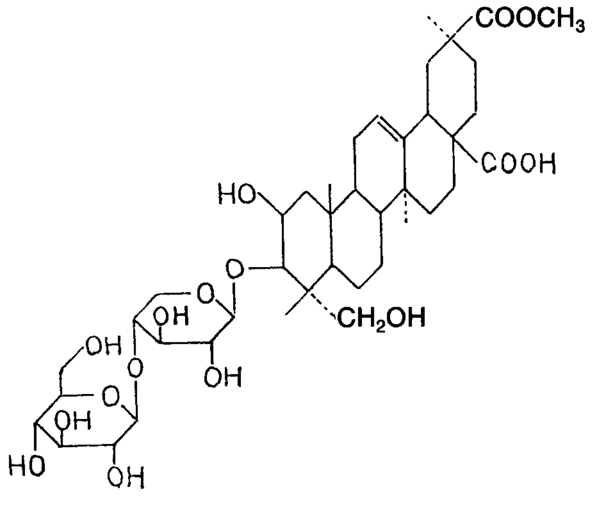

FIG. 1. The structure of esculentoside A.

### Materials and Methods

*Mice:* Inbred mice C57BL/6, aged 8–10 weeks were obtained from the Zoological Experiment Center of the Second Military Medical University.

*Reagents:* RPMI-1640, calcimycin (A23187) and lipopolysaccharide (*Escherichia coli* 055:B5) were purchased from Sigma, USA. Thioglycolate was supplied by Shanghai Biological Research Institute.

*Preparation of macrophages:* Thioglycolate medium (1.5 ml, 3%) was injected i.p. into the C57BL/6 mice. Four days later, the macrophages in the peritoneal cavity were harvested with D-Hanks solution, washed twice in RPMI-1640, and adhered for 2 h at 37°C in a $CO_2$ incubator, in 24-well plates at a concentration of $1 \times 10^6$ macrophages/well in 1 ml RPMI-1640 with 10% foetal calf serum.

*TNF production:* 2 h later, the medium containing non-adherent cells was decanted and the wells were rinsed twice with D-Hanks and twice with RPMI-1640. Then the adherent cells were incubated with A23187 (1 μmol/l) for 6 h. After incubation, the medium was discarded, and the cells were washed three times with RPMI-1640 to remove A23187. Fresh culture medium without serum was added to every well with lipopolysaccharide (LPS, 10 ng/ml) in presence or absence of EsA. The cultures were incubated for another 6 h at 37°C in a $CO_2$ incubator. The supernatants were harvested and centrifuged. The cell-free supernatants were collected, dialysed in PBS for 24 h, and stored at −20°C prior to activity assay. To determine the cell associated intracellular TNF activity, the cells remaining in the wells were covered with 1 ml of

© 1992 Rapid Communications of Oxford Ltd fresh culture medium, and frozen at −40°C. After thawing, the cells were sonicated, and the lysates were centrifuged to remove the particles, and stored at −20°C for activity assay.

*TNF production assay:* TNF assay was performed essentially as described by Kunkel[5] with minor modifications. Briefly, L929 cells (50 000/well) were dispensed into 96-well flat-bottomed microtitre plates in a volume of 0.1 ml/well. The following day, the cells were incubated for 18 h in the presence of 1 μg of actinomycin D and serial 1:2 dilutions of test sample. Media were then decanted, and the remaining cells in each well were stained with crystal violet for about 15 min, washed with tap water, and dried at 40°C. Absorbance of the cells in each well was read using a microenzyme linked immunosorbent autoreader. Units of TNF activity were defined as in Reference 5.

*Cell viability:* The trypan blue exclusion test was performed after 6 h incubation with or without EsA. Cell viability was also determined by measuring lactate dehydrogenase (LDH) activity in the cell-free supernatants according to the method of Beutle.[6]

### Results

*Effects of EsA on extracellular production of TNF from macrophages:* The extracellar production of TNF in the supernatant after being dialysed was assessed by the killing of L929 cells. One unit of TNF was defined as the reciprocal of the dilution of a preparation that results in 50% survival of the cells. The results are presented in Table 1. It is observed that the decrease of TNF production was significant at the concentrations of 1 and 10 μmol/l EsA.

**Table 1.** Effects of EsA on extracellular and cell associated TNF production by 1 × $10^6$ mice peritoneal macrophages stimulated with 10 ng LPS. Results, expressed as units/1 × $10^6$ cells, are means ± SD of a typical experiment

| | Extracellular (units/1 × $10^6$ Mo) | Intracellular (units/1 × $10^6$ Mo) |
|---|---|---|
| LPS | 105.9 ± 17.2 | 15.0 ± 1.7 |
| LPS + EsA | | |
| 0.01 μmol/l | 85.8 ± 9.4 | 13.6 ± 2.2 |
| 0.10 μmol/l | 79.4 ± 6.4 | 11.4 ± 1.7 |
| 1.00 μmol/l | 64.2 ± 5.6** | 10.6 ± 1.2** |
| 10.00 μmol/l | 58.5 ± 6.6** | 8.7 ± 0.9** |

** $p < 0.05$.

*Effects of EsA on intracellar production of TNF from peritoneal macrophages:* The results in Table 1 demonstrate that EsA reduced not only the extracellular production of TNF but also the intracellular production of TNF in a dose dependent manner. Intracellular TNF production is lower than that of extracellular production, and the concentration of EsA that significantly decreased TNF production is the same.

*Effects of EsA on the kinetics of extracellular and cell associated TNF production:* Peritoneal macrophages were cultured in LPS with or without EsA, and the supernatants were harvested at 2 h intervals. Extracellular and intracellular TNF activity can both be detected after 2 h exposure to LPS. Figure 2 shows that intracellular and extracellular TNF production were both inhibited in a time dependent manner by EsA at a concentration of 5 μmol/l.

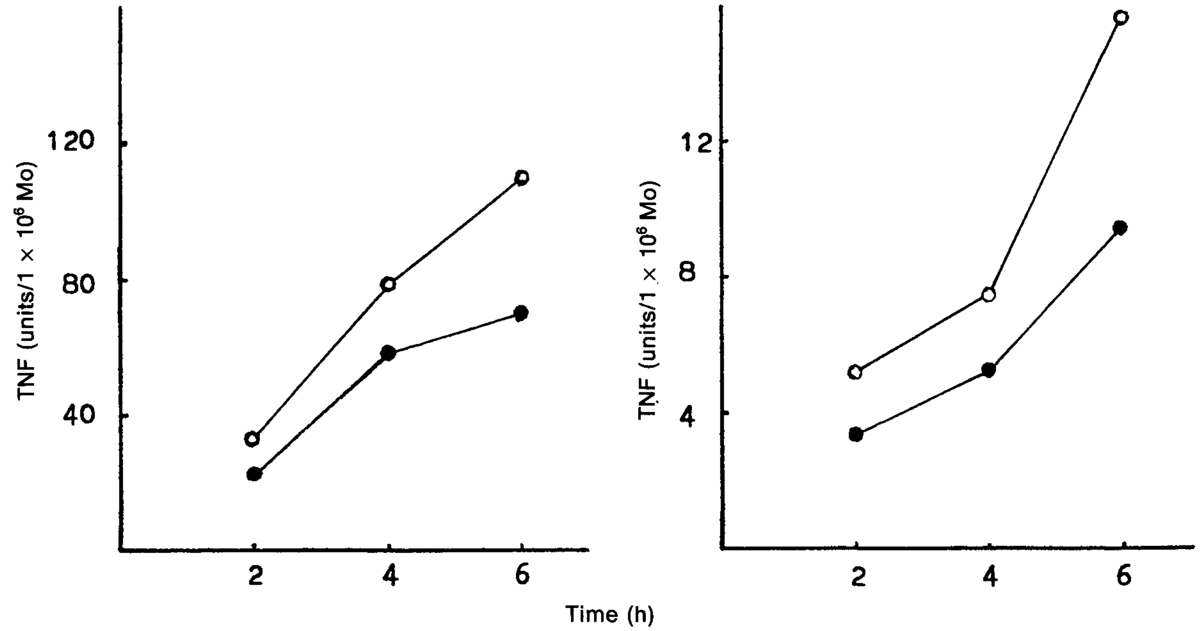

FIG. 2. Effect of EsA on the kinetics of TNF production in supernatants and lysates from macrophages cultures stimulated with 10 ng/ml LPS for 2, 4, 6 h, untreated (○), or treated (●), with 5 μmol/l of EsA. Similar results were obtained in two other independent experiments.

**Table 2.** LDH activities in supernatants from peritoneal macrophages incubated with or without EsA

| | LDH activity in supernatant (nmol NADH/min/1 × $10^6$ cells) |
|---|---|
| Control | $6.5 \pm 0.3$ |
| EsA | |
| 0.01 μmol/l | $6.7 \pm 0.2$ |
| 0.10 μmol/l | $6.4 \pm 0.1$ |
| 1.00 μmol/l | $6.8 \pm 0.3$ |
| 10.00 μmol/l | $6.8 \pm 0.1$ |

*Effects of EsA on cellular activity:* In order to eliminate the possibility that EsA was toxic for the cells tested, cell viability was monitored by measuring LDH activity in the supernatant and by the trypan blue exclusion test performed at the end of the macrophage culture. Results of the LDH in supernatant of adherent macrophages, cultured for 6 h with various concentrations of EsA, was not different from that of the control, as is shown in Table 2. Cell viability was confirmed by the trypan blue exclusion test (data not shown).

### Discussion

In this study, EsA induced a dose dependent decrease in the extracellular and cell associated TNF concentrations measured in the supernatant and lysates of LPS stimulated macrophages. The kinetics of extracellular and cell associated TNF production were also changed.

TNF was initially identified as a factor that appeared in the circulation of animals following the injection of endotoxins. TNF is a product of stimulated monocytes and macrophages, but it is also produced by keratinocytes.[7] In addition to the cytotoxic activities of TNF in some types of transformed cells, recent data have shown that TNF mediated stimulation of collagenase synthesis and prostaglandin E2 ($PgE_2$) production by synovial cells,[8] and stimulated bone resorption and inhibition,[9] suggesting that TNF might be an important mediator of inflammation. Platelet activating factor (PAF) is a mediator of anaphylaxis and inflammation; it plays an important role in inflammation, and there is cooperation between PAF and TNF in inflammatory reactions.[10] It has been found that EsA reduced release of PAF from rat macrophages. A previous study also showed that EsA can inhibit the inflammatory reaction induced by carrageenan.[1] Thus, together with the present investigation it is suggested that the anti-inflammatory effects of EsA could be due to its reducing effects on release of TNF and PAF.

### References

1. Zheng QY, Mai K, Pan XF. Antiinflammatory effects of esculentoside A. *Chin J Pharmacol Toxicol* (in press).
2. Dinarello CA, Mier JW. Lymphokines. *New Engl J Med* 1987; **317**: 940–945.
3. Maestre P, Zarco C, Guerrero CG. Cooperation between tumor necrosis factor (TNF) and platelet-activating factor (PAF) in the inflammatory response. *J Lip Med* 1990; **2**: s151–s159.
4. Fang J, Zheng QY. Inhibitory effects of esculentoside A on platelet activating factor released from calcimycin induced rat peritoneal macrophages. *Acta Pharmaceutica Sin* 1991; **26**: 721–724.
5. Kunkel SL, Spengler M, May MA, Spengler R, Larrick J, Remick D. Prostaglandin E2 regulates macrophages-derived tumor necrosis factor gene expression. *J Biochem* 1988; **263**: 5380–5384.
6. Beutle E. Lactate dehydrogenase. 2nd edn. New York: Grune and Stratton, 1975; 63–64.
7. Dinarello CA. Cytokines: Interleukin-1 and tumor necrosis factor (cachectin). New York: Raven Press, 1988; 54.
8. Dayer JM, Beutler B, Ceram A. Cachectin/tumor necrosis factor stimulates collagenase and prostaglandin E2 production by human synovial cells and dermal fibroblast. *J Exp Med* 1985; **162**: 2163–2168.
9. Bertolini DR, Nedwin GE, Bringman TS, Smith DD, Mundy GR. Stimulation of bone resorption and inhibition of bone formation *in vitro* by human tumor necrosis factor. *Nature* 1986; **319**: 516–518.
10. Braquet P, Braquet MP, Bourgaain QH, Bussolin F, Hosford D. PAF/cytokin auto-generated feedback networks in microvascular immune injury: consequences in shock, ischemia and graft rejection. *J Lip Med* 1989; **1**: 75–112.

ACKNOWLEDGEMENTS. We are grateful to H. S. Fang for typing this manuscript.

**Received 14 July 1992; accepted 20 August 1992**
